# 

**Published:** 1865-03

**Authors:** 


### BOOKS AND PAMPHLETS RECEIVED.

*Prospectus of the Detroit Preparatory School of Medicine, Detroit, Mich. Instructors*—Samuel P. Duffield, M. D., Ph. D., *Materia Medica, Chemistry and Texicology;* E. W. Jenks, M. D., Harper Hospital, *Obstetrics, Infantile Therapeutics, and Clinical Medicine;* D. O. Farrand, Ass't Surgeon U. S. A., in charge Harper Hospital, *Anatomy and Surgery;* George P. Andrews, M. D., St. Mary's Hospital, *Physiology, Principles of Medicine, and Microscopy. The first Term will comemnce the first Tuesday in April,* 1865, *and continue five months. Communications to be addressed to* George P. Andrews, *Secretary, No.* 79 *Shelby street, Detroit.*

*Medical Lexicon—A Dictionary of Medical Science; containing a concise explanation of the various subjects and terms of Anatomy, Physiology, Pathology, Hygience, Therapeutics, Pharmacology, Pharmacy, Surgery, Obstetrics, Medical Jurisprudence, and Dendistry; Notices of Climate, and of Mineral Water; formulæ for Officinal, Empirical, and Dietetic Preparations; with the Accentuation and Etymology of the Terms, and the French and other Synonymes; so as to constitute a French as well as English Medical Lexicon.* By Robley Dunglison, M. D., LL. D., *Professor of the Institute of Medicine, etc., in the Jefferson Medical College of Philadelphia. Thoroughly revised and greatly modified and augmented. Philadelphia:* Blanchard & Lea, 1865.

*The Transactions of the American Medical Association, instituted* 1847. *Vol. XV. Philadelphia,* 1865.

*Harvard University,* 1865 — *Medical Department — Summer Session, commencing March* 13, 1865.

